# Implementing group metacognitive therapy to improve mental health in NHS cardiac rehabilitation: the PATHWAY beacons study of adoption, adherence and data capture

**DOI:** 10.3389/frhs.2026.1793055

**Published:** 2026-03-26

**Authors:** Adrian Wells, Andrew Belcher, David Reeves, Patrick Doherty, Paul Wilson, Lora Capobianco

**Affiliations:** 1 Division of Psychology and Mental Health, School of Psychological Sciences, Faculty of Biology, Medicine and Health, School of Health Sciences, The University of Manchester, Manchester, United Kingdom; 2 Research and Innovation, Greater Manchester Mental Health NHS Foundation Trust, National Health Service Foundation, Manchester, United Kingdom; 3 NIHR School for Primary Care Research, Williamson Building, Manchester Academic Health Science Centre, The University of Manchester, Manchester, United Kingdom; 4 Jean McFarlane Building, Faculty of Biology Medicine and Health, Centre for Biostatistics, School of Health Sciences, The University of Manchester, Manchester, United Kingdom; 5 Department of Health Sciences, University of York, York, United Kingdom; 6 Division of Population Health, Health Services Research and Primary Care, School of Health Sciences, University of Manchester, Manchester, United Kingdom

**Keywords:** cardiac rehabilitation, cardiovascular disease, depression, anxiety, implementation, mental health, metacognitive therapy

## Abstract

**Aims:**

Anxiety, depression, and post-traumatic stress symptoms are common in cardiac rehabilitation (CR) patients. Group metacognitive therapy (MCT) alongside CR can significantly improve such symptoms compared to usual care. We aimed to conduct the first implementation study of group-MCT in NHS CR services. The objectives were: 1. Establish sites and assess levels of adoption; 2. Revise and pilot data capture via national auditing systems to assess MCT attendance and uptake; 3. Assess site-level MCT-adherence under roll-out conditions.

**Methods:**

A mixed-methods study evaluated implementation of group-MCT in routine care in CR services. Services across England were recruited as early adopters and staff were trained. The National Audit of Cardiac Rehabilitation (NACR) database was modified to collect and assess performance of group-MCT data capture. Five implementation outcomes were assessed; uptake and adherence, data-capture and quality, patient characteristics, site-level of adoption, and treatment adherence.

**Results:**

Twenty-six courses of group-MCT were delivered across six services, with an average of 4.3 courses per site and 131 patients receiving treatment. 82.4% of patients attended at least four sessions. Five services met all outcomes and were classed as green; one failed on one criterion and was rated amber. Data capture worked but with some minor discrepancies. Levels of intervention adherence were excellent, with high consistency across sites and time.

**Conclusions:**

We established six sites meeting our recruitment threshold and demonstrated satisfactory data capture on MCT attendance and uptake via national auditing systems. Five out of six sites met all adoption criteria. Site level adherence and compliance was excellent at 86.7%. Wider-scale adoption could improve access to evidence-based psychological therapy and enhance outcomes across the 188 CR-services in England.

## Introduction

Cardiovascular disease (CVD) is the most common non-communicable disease and contributes more than any other disease condition to morbidity and mortality worldwide (1, 2). In 2023, 64,217 patients attended the UK's cardiac rehabilitation (CR) service (3). Following a cardiac event, CR aids recovery, promotes healthy habits, reduces mortality and hospital readmissions, and improves quality of life (4, 5).

The psychological burden of CVD (6–9) is well known, with anxiety, depression, and post-traumatic stress disorder (PTSD) common in CR patients. Approximately 30% of CVD patients experience anxiety and depression (10), and 48% of CVD patients with anxiety or depression symptoms also experience clinically significant symptoms of PTSD (8). Mental health problems in CVD are associated with increased risk of mortality, decreased treatment adherence, poor lifestyle factors, poorer quality of life, higher healthcare costs and readmission, and poorer long-term psychological adjustment (2, 6–18).

Given the significant mental health burden associated with CVD, it is disappointing that psychological interventions to manage anxiety and depression in CVD have produced small and inconsistent results (19–24). A Cochrane review and meta-analysis of 35 randomised controlled trials totalling 10,703 people with CVD comparing psychological treatments [i.e., cognitive behavioural therapy (CBT), relaxation, meditation], but not including metacognitive therapy (MCT), with usual care reported small effects on anxiety (pooled SMD −0.24, 95% CI: −0.09 to −0.38) and depression (pooled SMD −0.27, 95% CI: −0.15 to −0.39) (22). Small effects and insufficient evidence quality underscore the need to develop more effective treatments and conduct more robust trials. A more recent Cochrane review and meta-analysis of 21 randomised controlled trials also included MCT in the analysis. The authors found psychological interventions resulted in a moderate reduction in depression compared with no psychological intervention (SMD −0.36, 95% CI: −0.65 to −0.06) and moderate reduction in anxiety compared to no psychological intervention (SMD −0.57, 95% CI −0.96 to −0.18) (23). The results of the more recent review appear larger than previous reviews, but the authors do not make a distinction between treatment modalities.

The gaps that currently exist in the routine provision of psychological care in CVD and limitations in effectiveness evident in the earlier literature led to the PATHWAY research programme (25–36), which set out to evaluate the efficacy of MCT delivered alongside CR. MCT (37) is an evidence-based therapy that is effective in treating anxiety and depression in patients in mental health clinics and can be more effective than approaches such as CBT (38). Unlike CBT, MCT focuses on regulating repetitive negative thinking rather than focusing on reality-testing the content of negative thoughts. MCT appears well suited to mental health applications in medicine because the negative thoughts reported by patients living with physical illnesses are often realistic and do not readily lend themselves to disputation methods that are a central part of CBT.

The NIHR-funded PATHWAY research program (25–36) supported a suite of interlinked studies aimed at exploring the psychological needs of CR patients and the attitudes of CR staff towards mental health in CR, to assess the fit of the MCT-based intervention. CR patients reported a wide range of worries and difficulty controlling these, leading to worry about worry itself (25), a process known as meta-worry in MCT (37). CR patients also reported that they are reluctant to discuss the content of their worries and coped by trying to analyse their problems or by seeking medical reassurance (25). However, the practitioner-focused studies showed that whilst CR staff recognised that patients had unmet psychological needs, they reported feeling uncertain about what to do and reported lacking the skills to discuss and address the psychological issues presented (39). The PATHWAY program went on to conduct four randomised controlled trials to pilot and then definitively test two MCT formats: a face-to-face group treatment and a home-based self-help treatment, each delivered alongside usual CR. Those studies demonstrated the feasibility and acceptability of MCT in the CR context and found that MCT substantially improved anxiety and depression outcomes compared with usual CR [SMD=0.52 (0.29–0.75), *p* < 0.011], with the results maintained at twelve months follow-up [SMD=0.33 (0.10–0.57), *p* = 0.01]. An incidental finding was that MCT also appeared to lower the incidence of psychological deterioration compared with that observed under CR alone (30).

With the potential of MCT to improve mental health in CVD, an important next step concerns transferring the intervention from research to routine clinical settings. One of the challenges in developing new treatments and leveraging their impact is successful implementation into practice. There are a multitude of factors that can facilitate or impede the use, uptake and eventual roll-out of new treatments, including maintaining treatment adherence/fidelity, development and use of data-capture mechanisms, service level feasibility, acceptability, and contextual factors such as legislation, policies, resources, and leadership values (40, 41). Unfortunately, such factors are often overlooked during implementation leading to unsuccessful efforts to introduce innovations (42).

The present study, PATHWAY-Beacons, aimed to evaluate a program to implement group-MCT in CR services in National Health Service sites in England and establish data-monitoring mechanisms and beacon sites to support subsequent roll-out and evaluation. We aimed to address the following primary questions:

1. What is the level of adoption of group-MCT at each Beacon site and across all sites combined?
2. What is the NACR database capture and monitoring quality for group-MCT?
3. What is the level of staff adherence to the intervention across sites and over time?

In addition, we explored how CR patients attending MCT might differ from those participating in CR-only in terms of demographic and health characteristics.

## Methods

### Study overview

The study was a mixed-methods study evaluating the implementation of group-MCT delivered as part of routine care in CR services. Please refer to the study protocol for full details (43). Ethical approval was not required here because the study was conducted as a service audit using NHS England's routine practice National Audit of Cardiac Rehabilitation (NACR) database. In addition to the aims reported in this paper, we conducted qualitative interviews with practitioners, commissioners, and service managers to understand service-level needs, barriers, and facilitators for implementation. Evidence from the qualitative findings will be published separately.

### Site recruitment

Site recruitment was time-limited and involved a two-week expressions of interest window, after which we aimed to select 4–6 sites that met the following inclusion criteria:

1. Registered with NACR and achieved minimum amber status on the British Association for Cardiovascular Prevention and Rehabilitation's (BACPR) key performance indicators in the most recent audit period.
2. The service agreed to deliver group-MCT to at least one group (in addition to a pilot training group).
3. The service agreed to release two CR staff members for three training days and to participate in qualitative interviews.
4. The service must not be participating in any other service evaluations.

### Intervention

Group-MCT (or MCT for short) is a brief group-based psychological treatment that reduces anxiety, depression, and PTSD symptoms in CR patients. The intervention is evidence-based and supported by a structured treatment manual. The treatment consists of six weekly sessions lasting approximately 90 min each delivered face to face at CR centres. Group-MCT aims to help patients improve control of worry, rumination, and attention and modify the metacognitive beliefs that maintain these unhelpful thinking patterns. There are eight major treatment techniques that are used across the six sessions, namely (1) formulation, (2) socialisation, (3) the Spatial Attentional Control Exercise, (4) detached mindfulness, (5) worry and rumination postponement, (6) modifying metacognitive beliefs about the uncontrollability and danger of worry and rumination, (7) a ‘helpful behaviours prescription’, and (8) individual treatment summaries. [See Wells (30) for further details of the intervention and its effectiveness]. For further details on the active treatment techniques see Wells (37). CR practitioners received two online training sessions lasting approximately 3.5 h each on two separate days. This was followed by supervised delivery of a pilot group. Following the pilot group, practitioners received a 3 h online review session to discuss treatment delivery experiences and review any challenges. The intervention has been reported in line with the TiDieR-Rehab (44) and GUIDE-Rehab (45) frameworks and the Tidier Checklist (46) is available in Supplementary Material S1.

### Group-MCT patient eligibility

Patients referred to CR who met the NICE recommendations for acute coronary syndrome (NG185) and heart failure (NG106) were offered MCT alongside routine CR. Patients could be offered group-MCT by a CR staff member at any time point during their CR programme as this fits with the patient-tailored model of CR. Depending on the patient's preference they may have received group-MCT prior to, in parallel, or after the other components of CR. Patients who declined MCT or who were not offered the intervention received routine CR only. In our previous trials we selected patients who scored above cut-off on the HADS, however this criterion was not implemented in the current study to widen uptake and render participation based on a patient-led decision of subjective need.

### Data sources and collection

#### Patient participation in MCT

Primary data on the numbers of patients attending each MCT course was collected by the practitioners at each site, including details of attendance at each individual course session. Alongside this, we explored the feasibility of collecting MCT attendance data via the NACR database. The NACR database is a national resource that routinely collects a range of demographic, clinical, functional, and physiological measures on each CR patient at entry into CR and at discharge. In partnership with the NACR, this database was amended in January 2023 to include a new data field to capture whether a patient attended MCT. We note however, that we were not able to collect comparative data on the number of MCT sessions each patient attended via NACR.

#### Demographic and health status measures

Data concerning demographic characteristics and health status for individual CR patients was obtained from the NACR database. The database includes two measures in particular that were used to characterise health status upon entry into CR:

*The Hospital Anxiety and Depression Scale* [HADS] (47) is a 14-item self-report questionnaire that measures symptoms of anxiety (7 items) and depression (7 items). Items are rated using a 4-point (0–3) scale, with higher scores indicating elevated distress. Scores for each subscale range from 0 to 21 and can be categorised as normal (0–7), mild (8–10), moderate (11–14), or severe (15–21). The NACR pays an annual licence fee for HADS so that all NHS-based CR programmes can use it free of charge.

*The Dartmouth COOP* (48) is a self-report measure designed to evaluate the functional abilities of medical patients. It consists of nine items covering various areas, such as physical function, daily activities, pain, social activities, social support, emotions, overall health, changes in health, and quality of life. Each chart includes text and illustrations to assist the user in responding. The responses are graded on an ordinal scale of 1–5, with one being the best score.

### Implementation outcomes

The success of the implementation program at each site was assessed in terms of the following five aspects of implementation:

1 Uptake and attendance at MCT: To assess the uptake of MCT within services, we utilised the primary data on MCT attendance collected from the Beacon sites to examine the numbers of patients starting and completing MCT, the numbers of courses delivered, and the average course size.
2 NACR database data capture and monitoring quality: Given that group-MCT was a new NACR dataset field, a comparison was made between this, and the primary attendance data collected from Beacon sites themselves, to check the ability of the field to accurately capture data on numbers of patients attending MCT.
3 Characteristics of MCT vs. CR-only patients: The NACR data routinely collected at the start of CR was used to describe the cohort of patients attending MCT and to compare their baseline characteristics to other patients at the study sites receiving CR only. The NACR dataset encompasses CR patients stretching back many years, hence, to produce comparable groups for analysis we restricted the sample to patients who could potentially have participated in the MCT courses. This was defined as patients who started CR on or after 1st November 2021 (approximately 1 year before the first MCT sessions were delivered) and who were still active CR patients one week prior to the first MCT session at their therapy site. We further excluded patients who started CR after recruitment into MCT at their site had ceased.
4 Criteria for successful adoption: Successful adoption of MCT was defined a-priori as the delivery within the study timeframe of two, 6-session group-MCT courses (including the pilot training course) with three to ten patients per group, with at least 60% attending a minimum of four sessions [four sessions has previously been adopted as a clinically effective dose of group-MCT (26)]. We utilised a traffic-light criterion to determine successful adoption as follows:
  • Green—achieved all of the following: (a) Delivered two 6-session group-MCT courses, with (b) at least three patients per group, and (c) with a combined total of at least 60% attending four or more sessions.
  • Amber—achieved one of the three criteria given above.
  • Red—achieved none of the criteria.
  Sites that achieved a green rating were classed as having fully met the criteria for successful adoption. Amber ratings were considered partial adoption, where amendments may be made to lead to full adoption, while red ratings were indicative of a failure to meet the requirements for adoption.
5 Treatment adherence: We assessed level of adherence to the treatment protocol using adherence checklists completed by the practitioners at the end of each session. The checklists consist of six to eight items representing the components to be delivered in specific sessions, covering the main techniques in the treatment manual. The number of treatment components completed, any components missed, and the reasons recorded were summarised to assess therapist adherence to the MCT manual. We also assessed variation in adherence levels across Beacon sites and any changes (consistency) in adherence over time.

### Quantitative analysis

Quantitative analysis was based on descriptive statistics only, including counts, means, percentages and graphs. Statistical inference testing was not applied (e.g., when comparing MCT vs. CR-only groups) as there was no hypothesis of zero differences and successful adoption was based on meeting pre-specified threshold values. All analysis was conducted using Microsoft Excel and Stata V16.

## Results

### Establishment of beacon sites

#### Site recruitment

A total of one hundred and thirty-six services from across England were invited to apply for the study based on their classification as green or amber on the NACR certification. Of these, nineteen services expressed interest in participating within the two-weeks invitation window, and twelve CR services submitted applications within the recruitment period. Seven further services showed interest; however, they stated that they could not formally apply due to staffing shortages or ongoing recovery from the impacts of COVID-19 on their operations. One service reported having a high proportion of non-English speakers, which prevented their participation since the treatment manual is not currently available in other languages.

**Figure 1:**
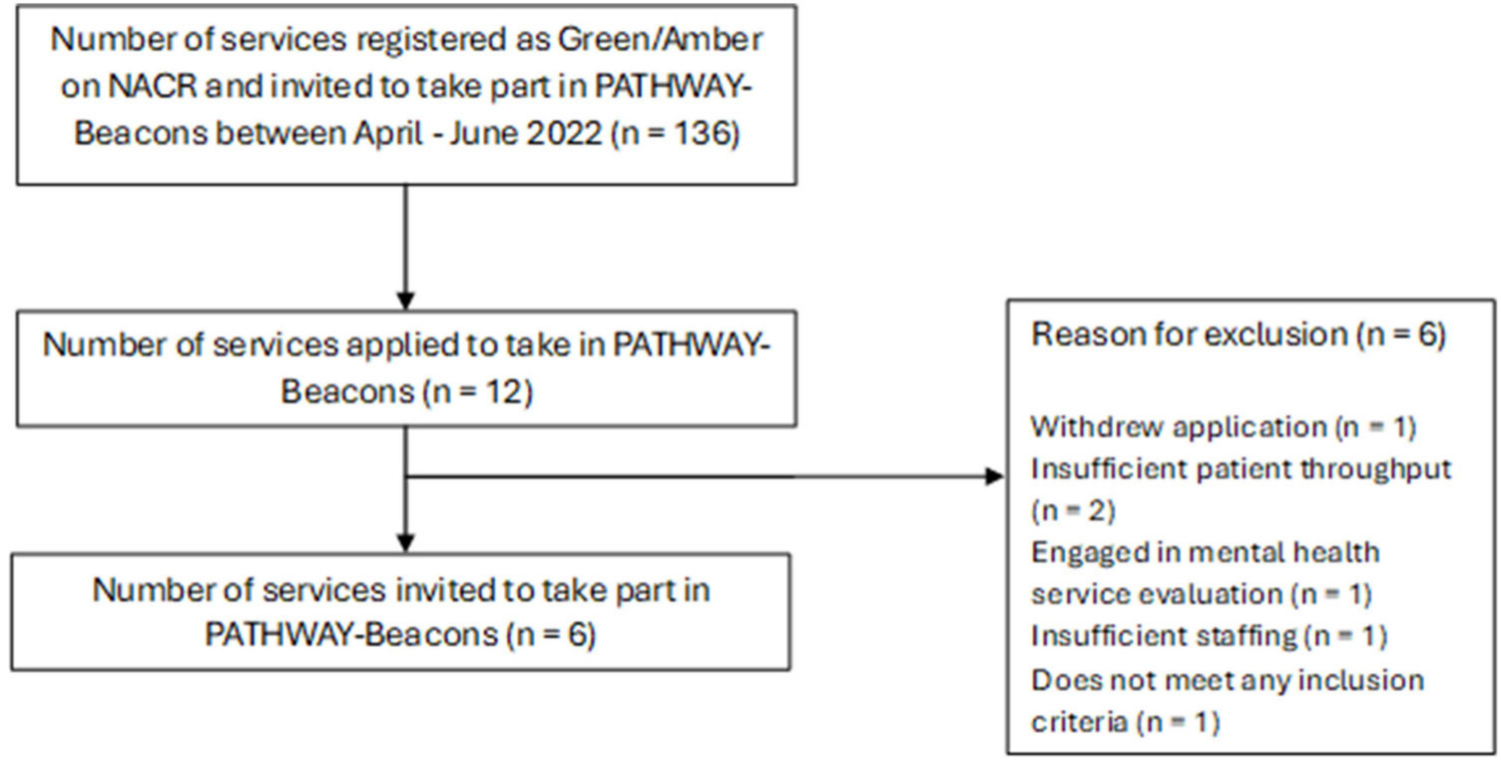
Beacon site recruitment flow diagram.

Twelve sites submitted applications to participate in the study but 6 of these sites were ineligible (Figure 1). Three sites were based in North-West England and represented services that had participated in the PATHWAY RCTs; additional services were situated in the Midlands, Yorkshire and Humber, and the South West of England. Based on NACR data, at all sites between 20% and 40% of CR patients exhibited clinically significant anxiety or depression; however, the study lacked services situated in the more affluent areas of the country. For more details about the included services, please refer to Table 1. See Figure 1 for a flow diagram illustrating site recruitment and why some interested services were excluded.

**Table 1:** Description of included beacon services.

CR Service	Geographic region	Hospital vs. community service	Socioeconomic region of service	No. of patients attending CR (in the previous year)	No. of patients who completed the HADS at CR assessment 1^a^	No. of patients with clinically significant HADS Anxiety score^b^	No. of patients with clinically significant HADS depression score^c^
A	North-West	Hospital	4th decile of IMD	314	262	94 (35.9%)	78 (29.8%)
B	North-West	Hospital	6th decile of IMD	429	161	48 (29.8%)	41 (25.5%)
C	Yorkshire & Humber	Hospital	5th decile of IMD	781	128	48 (37.5%)	35 (27.3%)
D	Midlands	Hospital	2nd decile of IMD	680	388	105 (27.1%)	80 (20.7%)
E	North-West	Community	1st decile of IMD	350	134	52 (38.8%)	48 (35.8%)
F	South-West	Hospital	6th decile of IMD	852	533	196 (36.8%)	156 (29.3%)

Data are number, *n* (%).

^a^ Clinically significant HADS score is denoted as a HADS score ≥8.

^b^ Number and percentages shown are only for patients who completed the HADS at assessment 1.

^c^ The number of patients who completed the HADS-D at Site D was 387. IMD, The Index of Multiple Deprivation; a measure of relative deprivation across the UK. Areas are ranked into deciles from the most deprived area (rank 1) to the least deprived area (rank 10).

### Adoption of MCT in CR services

#### Uptake and attendance at MCT

Attendance data collected at sites indicated that a total of 131 patients attended MCT as part of usual care, while a total of 26 courses of MCT were delivered across the six services (range 3–5 courses per service). The mean level of attendance was 4.8 sessions, with 82.4% of patients attending ≥4 sessions of group-MCT.

#### NACR database capture and monitoring quality

Of the 131 patients who received MCT, 123 were recorded in the new NACR dataset field as having started MCT (See Supplementary Material S1 for details), equating to a reporting accuracy of 93.9%. The eight-patient discrepancy in data reporting originated from three sites. In addition, other discrepancies were noted, involving six non-beacon sites that between them incorrectly coded eight CR-only patients as having participated in MCT. We examined the reasons for data discrepancies, summarised in the Supplementary Material S1.

#### Characteristics of MCT vs. CR-only patients

Out of the total 123 MCT cases recorded in the NACR dataset, six were dropped from the analysis: one with no recorded CR start date, two who started CR before 1 November 2021, and three who completed CR more than a week before the start of MCT. This left a sample of 117 MCT patients; the comparison group of CR-only patients meeting the same set of criteria consisted of 3,592 patients. The distribution of CR start dates was reasonably similar in the two groups.

Table 2 summarises the demographic and lifestyle characteristics of each treatment group. Patients in the MCT group tended to be a little younger, more likely to be female, of White ethnicity, and to live in areas of least deprivation. They were also distributed differently across the Beacon sites.

**Table 2:** Summary of demographic statistics for the MCT + CR and CR-only treatment groups.

Demographics	Group-MCT plus usual cardiac rehabilitation (*n* = 117)	Usual cardiac rehabilitation (*n* = 3,592)
Age at initiating event, years (mean, SD)	61.7 (10.2)	66.8 (12.0)
Gender (%)		
Male	70 (59.8)	2,548 (70.9)
Female	47 (40.2)	1,027 (28.6)
Not stated	0 (0.0)	17 (0.5)
Hospital site (%)		
A	21 (18.0)	1,049 (29.2)
B	24 (20.5)	531 (14.8)
C	16 (13.7)	761 (21.2)
D	23 (19.7)	199 (5.5)
E	13 (11.1)	692 (19.3)
F	20 (17.1)	360 (10.0)
Ethnicity (%)		
White	100 (90.1)	2,777 (86.3)
Mixed	0 (0.0)	17 (0.5)
Asian	6 (5.1)	180 (5.0)
Black	3 (2.6)	17 (0.5)
Other	0 (0.0)	23 (0.6)
Not stated	8 (6.8)	578 (16.1)
Rural-Urban classification (%)		
Rural	14 (12.0)	430 (12.0)
Urban	102 (87.2)	3,128 (87.1)
Unknown	1 (0.9)	34 (1.0)
Indices of multiple deprivation (%)		
1st quintile (most deprived)	26 (22.2)	774 (21.6)
2nd quintile	20 (17.1)	688 (19.2)
3rd quintile	18 (15.4)	674 (18.8)
4th quintile	18 (15.4)	760 (21.2)
5th quintile (least deprived)	33 (28.2)	650 (18.1)
Unknown	2 (1.7)	46 (1.3)
Employment (%)		
Economically active	29 (24.8)	787 (21.9)
Unemployed/Looking for work	6 (5.1)	57 (1.6)
Retired	22 (18.8)	1,492 (41.5)
Other	11 (9.4)	214 (6.0)
Not stated	49 (41.9)	1,042 (29.0)
Civil status (%)		
In relationship	36 (30.8)	812 (22.6)
Separated/widowed	3 (2.6)	96 (2.7)
Single	12 (10.3)	247 (6.9)
Not stated	66 (56.4)	2,437 (67.9)
Body-mass index (mean, SD)		
BMI	29.8 (6.0), *n* = 104	28.8 (6.0), *n* = 3,024
Smoking status (%)		
Never smoked	52 (44.4)	1,467 (40.8)
Ex-smoker	44 (37.6)	1,547 (43.1)
Current smoker	13 (11.1)	222 (6.2)
Not stated	8 (6.8)	356 (9.9)
Alcohol use (mean, SD)		
Units per week	4.5 (8.7), *n* = 84	5.9 (11.3), *n* = 2,129

Data are mean (SD) or number, *n* (%).

Table 3 presents group statistics on physical and psychological factors. These data show that the initiating event was slightly more likely to be a heart attack for patients in the MCT group (62% vs. 52%). However, a smaller percentage of the MCT group reported a previous cardiac event (31% vs. 43%). There were some group differences in the pattern of comorbid conditions, but both groups had an average of 2.1 comorbidities.

**Table 3:** Physical health and mental health summary statistics.

Health characteristics	Group-MCT plus usual cardiac rehabilitation (*n* = 117)	Usual cardiac rehabilitation (*n* = 3,592)
Initiating event (%)		
MI/Cardiac arrest	73 (62.4)	1,882 (52.4)
Angina	8 (6.8)	385 (10.7)
Unstable angina	2 (1.7)	63 (1.8)
Heart failure	9 (7.7)	326 (9.1)
Valve disease	6 (5.1)	369 (10.3)
CHD	1 (0.9)	83 (2.3)
Arrhythmia	1 (0.9)	16 (0.5)
Cardiomyopathy	2 (1.7)	20 (0.6)
Congenital heart disease	0 (0.0)	6 (0.2)
Prehab	0 (0.0)	1 (0.0)
Use treatment	9 (7.7)	309 (8.6)
Other	5 (4.3)	118 (3.3)
Unknown	1 (0.9)	14 (0.4)
Previous cardiac event (%)		
None stated	81 (69.2)	2,059 (57.3)
Yes	36 (30.8)	1,533 (42.7)
Type of previous cardiac event (%)		
MI/Cardiac arrest	12 (10.6)	417 (11.6)
Pacemaker	1 (0.9)	79 (2.2)
LV assist device	0 (0.0)	1 (0.03)
Angina	7 (6.0)	234 (6.5)
ICD	2 (1.7)	35 (1.0)
Bypass surgery	4 (3.4)	186 (5.2)
Other surgery	0 (0.0)	125 (3.5)
Congenital heart	0 (0.0)	9 (0.3)
Angioplasty	13 (11.1)	615 (17.1)
Heart failure	6 (5.1)	170 (4.7)
Transplant	0 (0.0)	1 (0.03)
Other	7 (6.0)	103 (2.9)
Unknown	1 (0.9)	188 (5.2)
Comorbidities^a^ (%)		
Angina	16 (13.7)	682 (19.0)
Osteoarthritis	8 (6.8)	445 (12.4)
Cancer	8 (6.8)	259 (7.2)
Diabetes	12 (10.3)	792 (22.1)
Rheumatism	7 (6.0)	58 (1.6)
Stroke	0 (0.0)	181 (5.0)
Osteoporosis	0 (0.0)	52 (1.5)
Hypertension	28 (23.9)	1,007 (28.0)
Chronic bronchitis	5 (4.3)	163 (4.5)
Emphysema	3 (2.6)	191 (5.3)
Asthma	5 (4.3)	257 (7.2)
Claudication	2 (1.7)	116 (3.2)
Back problems or chronic pain	6 (5.1)	175 (4.9)
Anxiety	12 (10.3)	110 (3.1)
Depression	6 (5.1)	135 (3.8)
Family History	15 (12.8)	285 (7.9)
Erectile Dysfunction	0 (0.0)	13 (0.5)
Hypercholesterolaemia/dyslipidaemia	24 (20.5)	473 (13.2)
Other illnesses	36 (30.8)	941 (26.2)
Total number of comorbidities (mean, SD)	2.1 (1.5) *n* = 94	2.1 (1.5) *n* = 2,959
Hospital Anxiety and Depression Scale (HADS) (mean (SD)		
Total score	17.3 (7.6) *n* = 76	11.3 (7.7) *n* = 1,687
Anxiety	9.9 (4.2) *n* = 77	6.0 (4.3) *n* = 1,688
Depression	7.4 (4.0) *n* = 76	5.3 (4.1) *n* = 1,689
HADS ≥ 8^a^ (%)	68 (58.1)	1,068 (29.7)
Dartmouth COOP total (mean, SD)		
Physical fitness	4.0 (0.9) *n* = 69	3.9 (1.1) *n* = 1,848
Feelings	3.2 (1.2) *n* = 71	2.3 (1.2) *n* = 1,861
Daily activities	3.0 (1.2) *n* = 70	2.5 (1.2) *n* = 1,855
Social activities	3.1 (1.3) *n* = 70	2.3 (1.3) *n* = 1,850
Pain	3.0 (1.1) *n* = 71	2.5 (1.2) *n* = 1,855
Change in health	2.6 (1.0) *n* = 71	2.2 (0.9) *n* = 1,857
Overall health	3.7 (0.9) *n* = 71	3.3 (1.0) *n* = 1,861
Social support	1.8 (1.1) *n* = 71	1.8 (1.2) *n* = 1,854
Quality of life	2.9 (0.8) *n* = 70	2.4 (0.8) *n* = 1,851
Total score	27.2 (5.8) *n* = 71	23.2 (6.2) *n* = 1,858

Data are mean (SD) or number, *n* (%).

^a^ The reported percentages are based on including cases with missing data in the denominator.

Completed HADS at baseline assessment were available for 65% of the MCT group (*n* = 76) but only 47% of the CR-only group (*n* = 1687). Amongst those who completed the form, mean HADS scores were higher in the MCT group for both the anxiety (mean 9.9 vs. 6.0) and depression (mean 7.4 vs. 5.3) subscales. Including those who did not complete the HADS in the denominator, 58% of the MCT group had a total HADS score of ≥8, compared to 30% of the CR-only group. Dartmouth COOP forms at baseline assessment were available for 61% (*n* = 71) of the MCT group and 52% (*n* = 1,861) of the CR-only group. Total COOP scores at assessment 1 were a little higher (indicating lower well-being) for the MCT group [27.2 (5.8) vs. 23.2 (6.2)].

#### Service adoption criteria

Table 4 displays that after applying the traffic light criteria for MCT adoption success at each service, five services met all three criteria and were classed as green. One other service was classed as amber because one course was delivered with less than three patients.

**Table 4:** Adoption of traffic light criteria.

CR Service	Adoption Outcomes			Adoption criteria			
	No. of MCT courses delivered	No. of patients attending group-MCT	Number of patients attending ≥4 sessions of group-MCT	Delivered at least two Group-MCT courses	Three patients per group	Patient attendance of at least 60%	Adoption traffic light criteria
Service A	5	23	20 (87.0%)	Yes (5 groups)	Yes	Yes (87.0%)	Green
Service B	5	26	22 (84.6%)	Yes (5 groups)	Yes	Yes (84.6%)	Green
Service C	4	17	14 (82.6%)	Yes (4 groups)	No	Yes (82.4%)	Amber
Service D	3	18	13 (72.2%)	Yes (3 groups)	Yes	Yes (72.2%)	Green
Service E	4	23	15 (65.2%)	Yes (4 groups)	Yes	Yes (65.2%)	Green
Service F	5	27	24 (88.9%)	Yes (5 groups)	Yes	Yes (88.9%)	Green

Data are number, *n* (%).

#### Staff training and treatment adherence

Thirteen CR staff (12 Female and 1 Male) were trained to deliver group-MCT. Twelve staff attended all training sessions; one member could not attend due to annual leave. The training sessions were recorded, allowing staff who missed them to complete them online. All sites and staff successfully delivered all six sessions of the pilot group for MCT, with full attendance from the staff members. All thirteen staff members attended the supervision and review of the pilot delivery.

Treatment adherence ratings were not returned for two groups (site A, group 5, and site F, group 3). One site (site C) did not deliver one session due to patients’ inability to attend, with 7% of the adherence data missing. Treatment adherence was high, with an average adherence rating across sites of 87.6% (range: 67.6%–100%). However, all deviations were due to non-completion of the adherence checklist, which occurred at four out of six sites, rather than a failure to deliver the treatment components *per se*. Non-completion of the adherence checklist occurred primarily in session six (*n* = 3), session four (*n* = 2), and session five (*n* = 1). Over time, the adherence rate ranged from 95% to 83% from the first to the last course of treatment.

## Discussion

This is the first study to assess implementation of MCT in CR services in the NHS. The study findings are encouraging and suggest that the adoption of group-MCT within NHS CR programs could be successfully achieved. Thirteen staff attended MCT training across six participating sites. Five Beacon sites successfully met all of our adoption criteria (designated as green). In contrast, one site was partially successful (designated as amber), as it did not recruit enough patients for one MCT course, although it did successfully recruit to three other courses. Overall, the adoption of group-MCT across CR services appears to be possible.

The patients attending MCT were similar to those attending only CR in terms of physical health, but those recruited to MCT reported higher levels of baseline anxiety and depression. We do not know the extent to which this contrast is due to patient choice or to recruitment practices amongst CR staff. Whilst we informed sites that all interested patients would be eligible to participate in MCT, site staff told us in qualitative interviews that they viewed some patients as “more in need” of psychological help, increasing the likelihood of some patients being approached to participate. Implementation will require service leaders and staff to consider whether the intervention is made available to all patients or primarily to patients considered to have a greater need for mental health support. Whilst such a decision is likely to be influenced by resource and cost considerations, it is important to question whether CR staff are currently trained and experienced enough to make decisions about the psychological needs of CR patients. In particular, we note from findings of previous studies that patients report not wanting to talk about their worries (26), and men may be more reticent in acknowledging their mental health needs (42), making detection of issues unreliable. One way to approach decisions about who is offered MCT is to set minimum qualifying criteria based on routine mental health assessments such as the HADS. In the PATHWAY trial (31) of group-MCT in CR, the sample was based on patients scoring at least eight on the anxiety or depression subscale of the HADS, a cut-off signifying the presence of clinical symptoms. Unfortunately, the completion rates for routine psychological outcomes (e.g., HADS) vary substantially across services, with an average return rate of 45% in 2020 (49). This raises several challenges as it will impact the ability to screen patients for access to MCT and to monitor the impact on psychological outcomes. An approach to overcoming such screening issues is to offer psychological awareness training to CR staff to increase knowledge and skills in detecting psychological issues and reinforce the value and use of screening tools.

An important consideration in the roll-out of effective treatment is the extent to which treatment is delivered as originally intended (adherence) and how consistent adherence is across sites and time. The overall adherence level in the present study was excellent (87.6%), whilst a low rating at one site (67.6%) reflected missing adherence data rather than a failure to deliver the treatment components. The adherence rate ranged from 95% to 83% from the first to the last course of treatment delivered, demonstrating stable adherence across the study period. These findings are promising and support the implementation of MCT protocol-based therapy in CR services.

Reliable data collection and monitoring of patient attendance and outcomes is necessary to evaluate and improve services. Implementation and roll-out of MCT in CR will need to be monitored, and data will need to be collected to assess the impact of MCT on health outcomes. One of the novel aspects of this study was our partnership with the NACR which enabled us to proactively add a data field to the national audit database to capture participation in MCT and compare this against the data captured separately at the site level. The NACR database was found to slightly under-record participation in MCT, by around 10%. We suspect the absent cases to principally be heart failure patients and those registered for National Data Opt-out, though we lack the patient-level data to confirm this. We also found a few instances where non-Beacon services had recorded CR-only patients as having participated in MCT, but the incidence rate was small and likely to become even smaller as services adjust to using the new field.

Limitations of the current study include a small number of participating Beacon sites (due to a narrow recruitment window) and the inability to offer the intervention to non-English speaking patients. Future research should aim to evaluate MCT in non-English speakers to reduce health inequalities. We do not know how many additional patients were offered MCT but turned the offer down; also, whether the practice of CR staff preferentially offering MCT to patients considered most likely to benefit, impacted attendance rates, adherence, and other adoption parameters that were assessed. The current inability to directly capture whether patients were offered group-MCT or the number of MCT sessions attended by patients via the NACR database (we can only detect if patients received group-MCT) means that future evaluation of attendance and effects of treatment dose is currently limited via this route. Further database modification would be required to capture more detailed attendance data if NACR is used as the sole data-capture mechanism for MCT going forward. Although the current preliminary findings are promising, they may be affected by bias. We did not use random sampling, and the volunteer sites may be more motivated and better resourced than other sites, which could enhance the implementation outcomes observed. We did not observe evidence of bias in the fidelity of the intervention, as adherence was high and consistent across sites, but the number of sites was small. The utilisation of an external national data-collection system may reduce measurement bias but consistent high-level utilisation of the data system across a wider number of sites needs to be explored.

In conclusion, the results of the present study supports wider roll-out and evaluation at scale of group-MCT in CR services in the National Health Service. The benefits of doing so include the incorporation of a standardised, evidence-based treatment to support the mental health of patients with CVD. Roll-out could address major gaps in the availability of routine mental health support in CR; it could improve mental health outcomes and improve CR staff's knowledge and skill levels in delivering much-needed multi-level interventions that improve physical and mental health outcomes in CVD patients.

## Data Availability

The datasets presented in this article are not readily available. The data was collected from The National Audit of Cardiac Rehabilitation (NACR) database. Further queries can be directed to the corresponding author.
